# Detection of *Mycobacterium bovis* in Bovine and Bubaline Tissues Using Nested-PCR for TbD1

**DOI:** 10.1371/journal.pone.0091023

**Published:** 2014-03-11

**Authors:** Cristina P. Araújo, Ana Luiza A. R. Osório, Kláudia S. G. Jorge, Carlos Alberto N. Ramos, Antonio Francisco S. Filho, Carlos Eugênio S. Vidal, Eliana Roxo, Christiane Nishibe, Nalvo F. Almeida, Antônio A. F. Júnior, Marcio R. Silva, José Diomedes B. Neto, Valíria D. Cerqueira, Martín J. Zumárraga, Flábio R. Araújo

**Affiliations:** 1 Programa de Pós-graduação em Ciência Animal FAMEZ, UFMS, Campo Grande, MS, Brazil; 2 Bolsista DTI, CNPq, Campo Grande, MS, Brazil; 3 PPGMV, UFSM, Santa Maria, RS, Brazil; 4 Instituto Biológico de São Paulo, São Paulo, SP, Brazil; 5 Faculdade de Computação, UFMS, Campo Grande, MS, Brazil; 6 Laboratório Nacional Agropecuário, Pedro Leopoldo, MG, Brazil; 7 Embrapa Gado de Leite, Juiz de Fora, MG, Brazil; 8 UFPA, Castanhal, PA, Brazil; 9 Instituto de Biotecnología, Instituto Nacional de Tecnología Agropecuaria, Buenos Aires, Argentina; 10 Embrapa Gado de Corte, Campo Grande, MS, Brazil; National Institute for Agriculture and Veterinary Research, IP (INIAV, I.P.), Portugal

## Abstract

In the present study, a nested-PCR system, targeting the TbD1 region, involving the performance of conventional PCR followed by real-time PCR, was developed to detect *Mycobacterium bovis* in bovine/bubaline tissue homogenates. The sensitivity and specificity of the reactions were assessed with DNA samples extracted from tuberculous and non-tuberculous mycobacteria, as well as other actinomycetales species and DNA samples extracted directly from bovine and bubaline tissue homogenates. In terms of analytical sensitivity, the DNA of *M. bovis* AN5 was detected up to 1.56 ng with conventional PCR, 97.6 pg with real-time PCR, and 1.53 pg with nested-PCR in the reaction mixture. The nested-PCR exhibited 100% analytical specificity for *M. bovis* when tested with the DNA of reference strains of environmental mycobacteria and closely-related Actinomycetales. A clinical sensitivity value of 76.0% was detected with tissue samples from animals that exhibited positive results in the comparative intradermal tuberculin test (CITT), as well as from those with lesions compatible with tuberculosis (LCT) that rendered positive cultures. A clinical specificity value of 100% was detected with tissue samples from animals with CITT- results, with no visible lesions (NVL) and negative cultures. No significant differences were found between the nested-PCR and culture in terms of detecting CITT+ animals with LCT or with NVL. No significant differences were recorded in the detection of CITT- animals with NVL. However, nested-PCR detected a significantly higher number of positive animals than the culture in the group of animals exhibiting LCT with no previous records of CITT. The use of the nested-PCR assay to detect *M. bovis* in tissue homogenates provided a rapid diagnosis of bovine and bubaline tuberculosis.

## Introduction

Tuberculosis is a chronic infectious disease caused by members of the *Mycobacterium tuberculosis* complex (MTC), including *M. tuberculosis*, *M. bovis, M. microti* [1], *M. africanum* [2], *M. canettii* [3], *M. caprae* [4], *M. pinnipedii* [5], *M. mungi* [6] and *M. orygis* [7]. In cattle, *M. bovis* is the species most frequently involved in tuberculosis cases. However other species of the MTC have been detected [7], [8], [9].

Bovine/bubaline tuberculosis causes financial losses in many countries and it is a zoonotic risk [10]. In Brazil, the control of bovine/bubaline tuberculosis is regulated by the Brazilian National Program for the Control and Eradication of Animal Brucellosis and Tuberculosis (PNCEBT). These regulations involve the slaughter of cattle with positive reactions to the intradermal tuberculin test (*ante-mortem* diagnosis) and the inspection of carcasses for gross lesions in abattoirs (*post-mortem* diagnosis) [11]. However, there is an increasing pressure from beef markets for a definitive diagnosis of tuberculosis in cattle exhibiting lesions compatible with tuberculosis (LCT). Since 2012, the Brazilian Ministry of Agriculture, Livestock and Food Supply (MAPA) determined that farms with cases of bovine/bubaline tuberculosis cannot export beef to the Customs Union of Belarus, Kazakhstan, and Russia. All lots of animals from a farm with suspicious animals are sequestered and the LCT are submitted to an official laboratory for etiological diagnosis [12].

The culture is considered to be the “gold standard” and definitive test for the confirmation of bovine tuberculosis. However, the microbiological diagnosis of *M. bovis* is an extremely slow procedure which may take as long as 2 to 3 months. Additional 2 to 3 weeks are required for the biochemical identification of isolates [13]. Therefore, the need for more rapid diagnostic systems is evident. Molecular diagnostic systems, particularly those based on real-time PCR technology, are faster [14].

The aim of the present study was to describe the development and assessment of a nested-PCR system, which involved a conventional reaction followed by real-time PCR, in terms of detecting *M. bovis* in bovine tissue homogenates.

## Materials and Methods

### Biological samples

Table 1 displays the reference bacterial strains used for the analytical sensitivity (*M. kansasii, M. smegmatis*, and *R. equi*) or specificity testing, as well as optimization of the nested-PCR. These include members of the MTC (*M. bovis* and *M. tuberculosis*), *Mycobacterium avium* complex (*M. avium*), atypical non-tuberculosis mycobacteria (*Mycobacterium abscessus, Mycobacterium fortuitum, Mycobacterium gordonae, Mycobacterium kansasii* and *Mycobacterium smegmatis*), and non-*Mycobacterium* Actinomycetales (*Corynebacterium pseudotuberculosis, Rhodococcus equi*).

**Table 1:** Bacterial standard strains used for evaluation of analytical specificity or analytical sensitivity of the nested-PCR.

Bacterial strains	Strain/origin
*Corynebacterium pseudotuberculosis*	LGCM/FIOCRUZ*
*Mycobacterium abscessus*	ATCC 19977/FIOCRUZ
*Mycobacterium avium*	ATCC 25291/FIOCRUZ
*Mycobacterium bovis*	AN5 strain, Agricultural Ministry – LANAGRO-MG
*Mycobacterium fortuitum*	ATCC 6841/FIOCRUZ
*Mycobacterium gordonae*	ATCC 14470/FIOCRUZ
*Mycobacterium kansasii*	ATCC 12478/FIOCRUZ
*Mycobacterium tuberculosis*	H37Rv/FIOCRUZ
*Mycobacterium smegmatis*	ATCC 700044/FIOCRUZ
*Rhodococcus equi*	ATCC 6939/FIOCRUZ

*Fundação Oswaldo Cruz, Instituto Nacional de Controle de Qualidade em Saúde.

The following materials were also used: a) 92 DNA samples obtained from cultures of lesions from cattle that had been naturally-infected with *M. bovis*. These samples were obtained from commercial cattle showing LCT during routine sanitary inspection in abattoirs, and lesions were submitted for etiological diagnosis at the Ministry of Agriculture – LANAGRO, MG, Brazil (n = 50) and *Instituto Biológico*, São Paulo, Brazil (n = 42), according to the regulation of the PNCEBT [11]; b) DNA from culture samples (n = 3) of *M. avium* from LANAGRO-MG. *M. bovis* and *M. avium* strains were identified using standard biochemical methods (samples from LANAGRO-MG) or PCR with the primers JB21 and JB22 for MTC [15]. Reference AN5 strain of *M. bovis* was cultured in Stonebrink medium, while the other *Mycobacterium* spp. reference strains were cultured in Löwenstein Jensen and Middlebrook media. Non-mycobacterium strains were not cultured and the DNA was purified directly from lyophilized bacterial suspension.

### Bacterial strains DNA isolation

DNA of reference bacteria was purified with DNEasy Blood & Tissue kit (Qiagen), following the manufacturer's instructions. The quality and concentration of the DNA samples were assessed by spectrophotometry (NanoDrop ND-1000, Thermo Scientific) and electrophoresis in 0.8% agarose gel, stained with SYBR Gold (Invitrogen).

### Primers and probes

Based on the DNA sequences of MTC members available at GenBank-NCBI (http://www.ncbi.nlm.nih.gov) and BLAST (http://blast.ncbi.nlm.nih.gov) searchers, specific targets were selected for DNA amplification of *M. bovis*. The primers and probe for nested-PCR were designed using Primer Express v2.0 software (Applied Biosystems).

The selected target for amplification was TbD1, a region that comprises the *mmpS6* gene (ID: 1092456) and the 5′ region of *mmpL6*. TbD1 is present in *M. bovis* (including BCG strains), *M. africanum*, *M. canettii*, and East-African Indian ancestral isolates of *M. tuberculosis*. In modern strains of *M. tuberculosis*, *mmpS6* is absent and *mmpL6* is truncated [03], [ 16, 17].

Two sets of primers were designed: outer primers, for conventional PCR amplification; internal primers and probe, for *TaqMan* MGB real-time PCR amplification. The first reaction was included to enrich *M. bovis* DNA, since the higher relative concentration of the host DNA isolated from cattle tissues may interfere with the amplification of the target gene. Sequences of the primers and probe are shown in Table 2.

**Table 2:** Primers and probes used for first conventional PCR and second real-time PCR for *Mycobacterium bovis* TbD1.

Target region	DNA sequences (5′→3′)
	Forward outer: GTGGCGGTCGCGGGATTCAGCGTCTAT
	Forward internal: GCGGTCTTCGCCAATGTT
TbD1	Probe: 6FAM TCGCGCAAGGCGA MGBNFQ
	Reverse internal: GCAGCCGATGGAATTGCT
	Reverse outer: TTATGGCGGCCACACCCACCCAAAACAG

### Nested-PCR standardization

Conventional PCR (first step) was carried out in a volume of 25 µl, containing 10 mM of Tris-HCl (pH 8.3); 50 µM of KCl; 1.5 mM of MgCl_2_, 0.2 mM of each dNTP, 7.5 pmol of each primer, 1.25 U of *Taq* DNA polymerase (Sigma), and 400 ηg of DNA.

Real-time PCR reactions (second step) for MTC were carried out in a volume of 12.5 µl, containing 6.25 µl of TaqMan Master Mix (ref 4352042, Applied Biosystems), 600 nM of each primer, 100 nM of the probe and 3 µl of the first step PCR reaction.

First step amplifications were carried out in an MJ Mini Biorad thermocycler. Initial denaturation was carried out at 95°C for 4 minutes, followed by 35 cycles of denaturation at 95°C for 90 seconds, annealing at 65°C for 30 seconds, and extension at 72°C for 45 seconds. A single 72°C final extension step was carried out for 3 minutes. Real-time PCR amplifications were carried out in a StepOne Plus thermocycler (Applied Biosystems, USA). Initial denaturation was carried out at 95°C for 10 minutes, followed by 35 cycles of denaturation at 95°C for 15 seconds and annealing/extension at 62°C for 30 seconds.

For all of the nested-PCR reactions, a positive control with DNA of *M. bovis* AN5, a blank control with no DNA and a negative control with DNA of H37Rv were included.

The primers/probe for *M. bovis* were tested for analytical specificity with 50 ηg of DNA of *C. pseudotuberculosis, M. abscessus, M. avium, M. fortuitum, M. gordonae, M. kansasii, M. smegmatis* and *R. equi*. Since TbD1 is absent in modern strains of *M. tuberculosis*, the strain H37Rv was also tested for PCR analytical specificity.

To test for PCR inhibitors, aliquots of DNA from the above species, used to assess specificity, were spiked with DNA of *M. bovis* AN5 and tested by nested-PCR. The amplification conditions were the same as those for the specific target.

The primers and probe were tested for analytical sensitivity with serial dilutions of DNA from reference strains of *M. bovis* AN5, in triplicate, but with one reaction mix for each replicate. The DNA samples were tested by real-time PCR singly, conventional PCR singly, and nested-PCR (both conventional and real-time reactions), and sensitivity was expressed as the minimum amount of DNA detected in the reaction mixture. In the case of nested-PCR, the volume of the first reaction (25 µl) was considered.

The primers and probe were also tested for analytical sensitivity with DNA extracted from 92 tissue cultures from naturally-infected cattle, provided by LANAGRO and the *Instituto Biológic*o.

### Direct detection of *M. bovis* in bovine and water buffalo tissues

Direct detection of *M. bovis* in tissue homogenates was carried out with 172 bovines and 62 water buffaloes (*Bubalus bubalis*) from commercial herds. No animals were slaughtered specifically for research. Tissue samples were collected during sanitary inspection in abattoirs from states of Amapá, Espírito Santo, Mato Grosso do Sul, Minas Gerais, Pará, Pernambuco, Rio Grande do Sul and São Paulo, Brazil, as follows:

- 127 comparative intradermal tuberculin test (CITT) positive animals, including 80 with lesions compatible with tuberculosis (LCT) and 47 with no visible lesions (NVL). These animals, from different age groups and zootechnical categories (dairy and beef cattle and water buffaloes), were culled following the recommendations of the PNCEBT [11].
- 51 CITT negative animals with NVL. These animals came from a mixed commercial farm (dairy and beef cattle and water buffaloes), with a previous history of tuberculosis.
- 51 animals with no records of CITT (dairy and beef cattle and water buffaloes) with LCT, detected during routine inspections in abattoirs.

Comparative intradermal tuberculin tests were conducted according to the regulations of PNCEBT [11]. A positive CITT reaction was defined as a relative increase in skin thickness at the injection site for bovine PPD of at least 4 mm greater than the increase in skin thickness at the injection site for avian PPD [11].

LCT were obtained for the present study from hepatic, iliac, mandibular, mediastinal, mesenteric, pre-scapular, retropharyngeal and tracheobronchial lymph nodes or the lungs, tonsils, liver or diaphragm. When cattle exhibited NVL, the hepatic, mediastinal, mesenteric, retropharyngeal and tracheobronchial lymph nodes were collected. The organs were kept on ice until they reached the laboratory, where they were kept at −30°C until processing. The organs were thawed and divided into two samples, one for culturing and the other for DNA isolation.

For DNA isolation, the samples were cut into pieces of 100 mg, corresponding to the transition between gross lesions and apparently healthy areas. These pieces were completely homogenized with 1 ml of phosphate buffered saline (PBS). From these tissue suspensions, 200 µl was used for DNA isolation with the DNEasy Blood & Tissue kit (Qiagen), following the manufacturer's instructions. Nested-PCR reactions were carried out as described above.

For culturing, the samples were thawed and homogenized with an equivalent amount of sterile sand and saline. The tissue suspensions were filtered through sterile gauze and centrifuged at 1200 g for 15 minutes. The sediments were suspended with 2 ml of sterile saline, decontaminated using Petroff's method [18], and cultured on the Stonebrink medium. The cultures were incubated at 37°C and searched for bacterial colonies for at least 90 days, with weekly observations. The smears from the isolated colonies were stained using the Ziehl-Neelsen method (ZN) for acid-fast bacilli (AFB). All the AFB cultures were analyzed by PCR, with primers JB21 and JB22, for MTC [15].

Cattle were considered positive for tuberculosis when at least a single tissue sample exhibited amplification in the nested-PCR or exhibited AFB cultures confirmed by the PCR with primers JB21 and JB22. Cattle were considered negative for tuberculosis when the cultures exhibited no AFB, when AFB cultures were negative for MTC in the PCR with primers JB21 and JB22, and when the tissues were negative in the nested-PCR.

### Statistical analysis

The agreement between the nested-PCR and the culture was assessed by the Kappa index [19]. Paired comparisons were performed using McNemar's test, with the level of significance set at 0.05. The chi-square test and Fisher's exact test were carried out to assess associations between the categorical variables, with the level of significance set at 0.05. The chi-square for linear trend was also used and, whenever possible, univariate logistic regression was performed to assess whether there was a dose-response relationship between the levels of presumptive evidence for bovine TB. Based on a combination of both diagnoses (CITT and LCT) and the result of the nested-PCR for each level of “exposure,” an odds ratio was calculated to compare the proportion of positive results with the baseline level or level 0 (no visible lesions and negative CITT) [20].

## Results

*In silico* analysis of the DNA primer and probe sequences exhibited complete identity with TbD1 sequences of *M. bovis* AF2122/97, different *M. bovis* BCG strains, *M. africanum* and *M. canettii*, available in the NCBI. Complete identity was also found for 7 strains of *M. bovis* from Argentina (including 04-303, accession number AVSW00000000), 10 strains of *M. bovis* from Brazil, and AN5 strain (accession number AWPL00000000), the genomes of which were sequenced by our group in other study. Regarding *M. tuberculosis*, the primers and probe exhibited complete identity with an ancestral strain (accession number AJ426486) [03], but no homology was detected with modern strains of *M. tuberculosis* available at GenBank.

No amplification was recorded in the following cases: DNA from the H37Rv strain or from clinical isolates of *M. tuberculosis*; DNA from reference strains of the non-tuberculous mycobacteria *M. abscessus, M. avium, M. fortuitum, M. gordonae, M. kansasii* and *M. smegmatis*; DNA from closely-related Actinomycetales *C. pseudotuberculosis*; *R. equi*. DNA aliquots of non-target microorganisms spiked with the DNA of *M. bovis* AN5 were positive in the nested-PCR.

With regards to analytical sensitivity, DNA of *M. bovis* AN5 was detected in the reaction mixture up to 1.56 ηg with conventional PCR, 97.6 pg with real-time PCR, and 1.53 pg with nested-PCR. Of the 50 DNA samples isolated from cultures from lesions of cattle that had been naturally-infected with *M. bovis* from the Ministry of Agriculture – LANAGRO, 49 (98.0%) were positive in the nested-PCR for *M. bovis*. All of the 42 (100%) DNA samples isolated from cultures of *M. bovis* from the *Instituto Biológico*, were positive in the nested-PCR for *M. bovis*.

Tissue samples from 229 bovines/bubalines were tested directly by nested-PCR for *M. bovis*. The nested-PCR and culture results are shown in Table 3. Aliquots of all the samples that showed negative results in the nested-PCR were spiked with DNA of *M. bovis* AN5 strain and re-tested in the nested-PCR, showing positive results. Of the 51 animals with CITT- results and NVL, 50 exhibited negative cultures. These 50 animals were included for the calculation of clinical specificity, which was 100%. Of the 50 animals with CITT+ results and LCT that rendered positive cultures, 38 were also positive in the nested-PCR, corresponding to a clinical sensitivity of 76.0%.

**Table 3:** Nested-PCR for *Mycobacterium bovis* TbD1 and culture results of 229 bovine and bubaline tissue homogenates.

Status			Test			Odds ratio**	
Ante-mortem	Post-mortem	Total Number	Culture* (%)	Nested-PCR (%)	P-value	Culture	Nested-PCR
CITT-	NVL	51	1 (1.96)a	0 (0.00)a	1.0	1.0A	1.0A
No CITT	LCT	51	11 (21.57)a	21 (41.18)b	0.0329	13.75B	72.61B
CITT+	NVL	47	11 (23.40)a	17 (36.17)a	0.176	15.27B	59.10B
CITT+	LCT	80	50 (62.50)a	49 (61.25)a	0.8707	83.33C	161.86C
	Total	229	73 (31.88)	87 (37.99)	0.17	-	-

CITT = Comparative intradermal tuberculin test.

LCT = Lesions compatible with tuberculosis.

NVL = No visible lesions.

*Confirmed by PCR with primers JB21 and JB22 [15].

**p-value for chi-square of linear trend <0.001.

Different lowercase letters in rows indicate significant differences (p<0.05) within the paired comparisons between culture and nested-PCR.

Different capital letters in the columns indicate proportions significantly different (p<0.05).

With regards to the agreement between the nested-PCR and the culture, there were 51 nested-PCR+/culture+ animal (22.27%), 120 nested-PCR-/culture- animals (52.40%), 36 nested-PCR+/culture- animals (15.72%) and 22 nested-PCR-/culture+ animals (9.61%). The agreement assessed by the Kappa index was 0.445, with a standard error of 0.061 and a 95% confidence interval from 0.325 to 0.565. The strength of the agreement is considered to be moderate [19].

Results from the nested-PCR and cultures revealed no significant differences in terms of detecting CITT+ animals with LCT or with NVL. No significant differences were found in the detection of CITT- animals with NVL. However, the nested-PCR detected a significantly higher number of positive animals than the culture in the group of animals with no records of CITT, but exhibiting LCT (p<0.05).

There was a linear trend between increasing presumptive evidence of bovine tuberculosis and the chance of positive results in the culture (p<0.001) or in the nested-PCR (p<0.001). The presumptive results of LCT or positive CITT produced 13.75 and 15.27 times more chance of a positive result in the culture, and 59.10 and 72.61 times more chance of a positive result in the nested-PCR, respectively, when compared with the group with no presumptive results. In the group with the two presumptive positive results, LCT and CITT produced 83.33 and 161.86 times more chance of a positive result in the culture and in the nested-PCR, when compared with the group with no presumptive results, respectively (Table 3).

## Discussion

One of the most essential systems applied to the eradication of bovine tuberculosis by *M. bovis* is the epidemiological surveillance of animals slaughtered in abattoirs. This surveillance is conducted by means of inspection and taking samples of LCT, confirming the existence of the disease through the culture and molecular detection, which takes weeks before a result can be obtained [21].

The aim of the present study was to develop a *post-mortem* diagnostic system for bovine and bubaline tuberculosis, applicable directly to bovine clinical samples, that could substantially reduce the time between the detection of CITT+ animals and/or LCT and the etiological diagnosis, when compared to the traditional method of culturing, which takes up to 90 days.

A nested-PCR system targeting the TbD1 was developed. This region comprises the *mmpS6* gene and the 5′ region of *mmpL6*, which are present in the genomes of *M. bovis*, *M. africanum, M. canettii* and in ancestral strains of *M. tuberculosis* [03, 22]. As expected, BLASTn analysis of the sequences of the primers and probe for TbD1 exhibited complete identity with the above MTC species. However, there is little genomic information about field strains of *M. bovis* to assess the conservation of the target sequences of the nested-PCR. Our research group sequenced the genomes of 7 strains of *M. bovis* from Argentina and 11 strains from Brazil, and target sequences were conserved in all 18 strains.

The analytical specificity of the nested-PCR was analyzed *in vitro*. No amplification was detected when the primers/probe for TbD1 were used with DNA of *M. tuberculosis* H37Rv or from cultures of clinical isolates of *M. tuberculosis*. However, only H37Rv was tested in the present study and this is a modern genotype of *M. tuberculosis* [23].

There are reports of *M. tuberculosis* infecting cattle worldwide [24], [25], [26], [27]. In Brazil, more than 2000 culture samples from cattle with presumptive lesions of tuberculosis were tested for *M. tuberculosis* at LANAGRO-MG (Brazilian Ministry of Agriculture) and no positive results were found (A. A. Fonseca Júnior, 2013, personal communication).

In humans, modern genotype strains (TbD1 deleted) of *M. tuberculosis* are most commonly found in Morocco, Cameroon, Ethiopia, Pakistan, Myanmar [17], [28], [29], [30], [31]. However, in Bangladesh, 65% of the *M. tuberculosis* isolates were ancestral (TbD1+) [32]. In India, modern genotypes are predominant in the south of the country, whereas ancestral genotypes are more common in central and northern areas of the country [33].

No amplification was detected with the primers/probe for TbD1, DNA of the environmental mycobacteria *M. abcessus, M. avium, M. fortuitum, M. gordonae, M. kansasii* and *M. smegmatis*, or with the DNA of the closely-related bacteria tested. This is particularly significant since environmental mycobacteria present in lymph nodes submitted for diagnostic testing can confound assays that lack sufficient specificity [34]. Furthermore, closely-related Actinomycetales, such as *R. equi* and *C. pseudotuberculosis*, may cause lesions to be mistakenly diagnosed as tuberculosis [35], [36].

Nested-PCR for TbD1 was not tested with the DNA of *M. africanum* or *M. canettii*, which are TbD1 positive. However, both species are essentially human pathogens [03, 37] and have not been described in Brazil. A number of reports of *M. africanum* in cattle have been described in Europe and Asia [08], [ 38, 39]. The epidemiological importance of this pathogen in cattle must be clarified, especially in new world countries.

Traditionally, clinical sensitivity and specificity for PCR reactions to tuberculosis are assessed with tissue samples that are positive or negative, respectively, in the culture for *Mycobacterium* sp., since the culture is considered to be the gold-standard diagnostic test for tuberculosis [21]. In this sense, nested-PCR exhibited a moderate clinical sensitivity and a high clinical specificity. Nevertheless, in terms of the use of an additional diagnostic tool as part of a control/eradication program of bovine tuberculosis, this information is limited. Depending on the stage of the infection or even the condition of the biological sample, PCR may detect more animals than the culture [21], or vice-versa [40], [41]. Feasibility is another important factor, since carcasses with LCT in abattoirs require rapid diagnostic results.

In the present study, nested-PCR detected 36 nested-PCR+/culture- animals, which corresponded to 15.72% of the number of samples tested. Additional suggestions that these animals were really infected include the presence of other presumptive evidence of tuberculosis in all animals (positive CITT: 26 animals; LCT: 21 animals; CITT and LCT: 11 animals). On the other hand, there were 22 culture+/nested-PCR- animals (9.61%). Since different methods of DNA purification from tissue may influence the presence of PCR inhibitors in the sample being tested [42], DNA aliquots from tissues of these 22 culture+/nested-PCR- animals were spiked with DNA from *M. bovis* AN5 strain and were re-tested by nested-PCR, showing positive results.

In Brazil, skin tuberculinization is the official *ante-mortem* test for bovine/bubaline tuberculosis. CITT+ animals are considered positive for tuberculosis and must be slaughtered in a maximum of 30 days [11], preferably in an official abattoir in which tissue samples are to be taken for microbiological diagnosis. Nested-PCR for TbD1 and the culture detected similar numbers of CITT+ animals (P = 0.5304). No statistically significant differences were found between nested-PCR and the culture in the numbers of CITT- animals detected.

During meat inspection at abattoir level, the main concerns are animals exhibiting LCT, which are considered inappropriate for consumption. However, in the bovine/bubaline samples of the present study, 37% of the CITT+ animals exhibited NVL, of which 14.9% were positive both in the culture and in the nested-PCR. This raises concerns that zoonotic transmission, such as that of *M. bovis*, can survive the cooking process [43]. For this reason, sanitary policies involving PCR testing of CITT+/NVL animals should be considered.

In Brazil, carcasses that exhibit LCT during routine sanitary inspections are condemned for consumption. Some countries that import meat from Brazil are imposing the requirement of an etiological diagnosis of such lesions. The nested-PCR was more sensitive than the culture when detecting *M. bovis* in animals that were not tested by CITT, but exhibited LCT during sanitary inspections at abattoirs. One of the possible reasons for this result is that this group included 23 water buffaloes from the Amazon region (State of Amapá), whose tissues were transported for a long period in high temperatures. Despite being stored in ice, the samples started to deteriorate, resulting in negative cultures, although the nested-PCR reactions were positive.

Several methodologies have previously been used to increase the sensitivity of real-time PCR when detecting *Mycobacterium* sp. directly from tissue homogenates. Parra et al. [21] used a capture probe to isolate mycobacterial DNA from tissue homogenates, achieving a lower sensitivity (65.6%) than that found in the present study. Taylor et al. [40] also reported a sensitivity of 70% when performing PCR directly on tissue homogenates, although the sensitivity increased to 91% when PCR was only performed on DNA isolated from lesions excised from the tissues rather than whole tissue homogenates. The limitation of this method is that the assay can only be performed on tissues that have LCT, thus excluding samples without readily apparent lesions. Thacker et al. [34] used a strategy that was similar to the present study, with conventional PCR followed by a real-time reaction, but targeting IS*6110*, and found a sensitivity of 66.7% in detecting positive culture samples.

One of the concerns about nested-PCR, particularly in relation to real-time reactions, is the possibility of cross-contamination. Throughout the DNA extraction procedure, gloves were changed frequently. DNA purification was carried out at biosafety level 3, with the PCR set-up on a laminar flow PCR cabinet with UV light. Separate sets of micropipettes were used for DNA purification and PCR set-up. Filter tips were used routinely. Surfaces and equipment in contact with sample tubes were cleaned before each assay. Blank and negative controls were always used.

Nested-PCR allowed the identification of *M. bovis* in tissues with a performance that was similar or superior to the culture. Individual results from the nested-PCR were obtained in a short period of time (2 days), in contrast with the culture, which took up to 90 days. This suggests that nested-PCR can be used for *post-mortem* etiological diagnoses of bovine/bubaline tuberculosis during routine inspections at abattoirs. However, a large-scale study and an inter-laboratory validation of the method are required to determine whether the method is adequate for the PNCEBT.

Finally, there was a linear positive trend, confirming a dose–response relationship between the increase of presumptive results, both in terms of the negative level (CITT – and NVL) and the positive level (CITT + and LCT), as well as the odds ratio for positive results in the culture and the nested-PCR. Dose-response and trend analysis are commonly employed in epidemiology and public health. In the present study, the dose-response was shown to be useful in terms of demonstrating the logic of laboratory results with the clinical data of the animals and assisting the decision-making process in diagnostic tests related to animal health surveillance and food inspection.

## Supporting Information

Table S1 Identification of isolates of Mycobacterium bovis by origin and abattoir, and results of culture and nested-PCR.
(XLS)
